# Retraction Note: Kynurenine promotes neonatal heart regeneration by stimulating cardiomyocyte proliferation and cardiac angiogenesis

**DOI:** 10.1038/s41467-025-57248-0

**Published:** 2025-03-03

**Authors:** Donghong Zhang, Jinfeng Ning, Tharmarajan Ramprasath, Changjiang Yu, Xiaoxu Zheng, Ping Song, Zhonglin Xie, Ming-Hui Zou

**Affiliations:** Center for Molecular and Translational Medicine, Georgia State University, 157 Decatur Street North East, Atlanta, GA 30303 USA

Retraction to: *Nature Communications* 10.1038/s41467-022-33734-7, published online 26 October 2022

The Editors have retracted this article after the following concerns were raised:

- The top left-hand panel of Figure 2e overlaps with the bottom right-hand panel of Figure 5e
- The top left-hand panel of Figure 2l overlaps with the bottom left-hand panel of Figure 5e

An investigation by Georgia State University concluded that these partially overlapping images came from two different fields under the microscope in one single slice. The Editors therefore no longer have confidence in the results and conclusions presented. Donghong Zhang disagrees with this retraction. Zhinglin Xie and Ming-Hui Zou agree with this retraction. Tharmarajan Ramprasath and Ping Song have not responded to correspondence from the Editors about this retraction. The Editors have not been able to find current email addresses for Jinfeng Ning, Changjiang Yu and Xiaoxu Zheng.

